# Acclimation to higher temperature and antioxidant supplemented diets improved rainbow trout (*Oncorhynchus mykiss*) resilience to heatwaves

**DOI:** 10.1038/s41598-024-62130-y

**Published:** 2024-05-18

**Authors:** Fatemeh Hosseinpour, Arya Vazirzadeh, Ahmad Farhadi, Seyed Hossein Sajjadi

**Affiliations:** Department of Natural Resources and Environmental Engineering, School of Agriculture, Shiraz University, Shiraz, Iran

**Keywords:** Physiology, Climate-change ecology

## Abstract

Coldwater species are challenged with increasing water temperatures and fluctuations over their upper thermal limits. This study evaluated the potential of acclimation to higher temperature and dietary antioxidants capacity to mitigate the adverse effects of heat shocks in rainbow trout. To this end, rainbow trout fingerlings were acclimated at optimal (14 °C) and high (20 °C) temperatures and fed on selenium (5 mg/kg) and polyphenol (2 g/kg) supplemented diets for 60 days and then were exposed to heat shocks by increasing water temperature up to 30 °C. Growth performance, survival rate, haemato-immunological parameters, and expression of *HSP70α*, *HSP70β*, *HSP90β,* and *IL-1β* genes were measured to evaluate the hypothesises. The rainbow trout acclimated to 20 °C and fed on antioxidants supplemented diets showed a significantly higher aftershock survival rate. Moreover, fish acclimated to higher temperature showed higher red blood cell counts as well as serum total protein and albumin during the acclimation trial and heat shocks phase. Acclimation to higher temperature and feeding on antioxidants remarkably enhanced fish immune and antioxidant capacity in comparison to fish adapted to cold water and fed on the basal diet measured by improved respiratory burst and lysozyme activities and upregulation of *IL-1β* expression during exposure of fish to heat shocks. Furthermore, fish acclimated to higher temperature, especially those fed on antioxidant supplemented diets, showed lower expression levels of *HSPs* genes during the heat shock phase, indicating that high heat shocks were less stressful for these fish in comparison to cold water acclimated fish. This finding was also supported by lower cortisol levels during heat shocks in fish acclimated to higher temperature. In conclusion, the results of this study indicated that acclimation to higher temperature and/or fed on diets supplemented by selenium and polyphenol, can help to mitigate the adverse effects of the heat shock in rainbow trout.

## Introduction

Climate change is affecting the quantity and quality (temperature, salinity, dissolved oxygen concentration, and pH) of water resources used in aquaculture^1–4^. Sub-optimum water quality and fluctuations in physicochemical parameters of water used in aquaculture frequently lead to compromised immune systems and increased mortality in cultured species^2,4^. Increasing and greater fluctuations in water temperatures associated with climate change are already adversely impacting the production of cold-water aquaculture species, especially salmonids, with major mortality events due to thermal stress reported in many regions of the world with salmonid aquaculture^5–7^. It has been reported that acclimation of salmonids to higher temperatures can considerably ameliorate some of the negative effects through a variety of adaptive metabolic responses^2,8,9^. For example, marked changes in gene expression at elevated temperatures in Pacific (*Onchorhynchus tshawytsha*) and Atlantic salmon (*Salmo salar*) support increased chaperoning and protein rescue via increased production of heat shock proteins (HSPs) and upregulation of some biosynthesis of proteins and steroid precursors^10,11^.

In addition, studies have already shown that dietary antioxidants and mineral supplements can play an important role in stimulating immune parameters and the tolerance of environmental stressors in fish^12–14^. Selenium is a micronutrient with vital roles in fish growth, maintenance of physiological functions and response to stress^15,16^. Selenium also plays a key role in the functional structure of several proteins (i.e., so called selenoproteins), with wide biological functions, such as preventing oxidative damage, maintaining homeostasis, and increasing immune function^15–17^. Organic forms of selenium, such as selenomethionine and selenium yeast, are shown to be more efficient for dietary uptake in fish than the mineral forms^15–19^.

Polyphenol extracted from chestnuts is a natural plant antioxidant which, with dietary provision, has been shown to improve growth performance and the immune system in fish species such as common carp (*Cyprinus carpio*) and bream (*Megalobrama amblycephala*)^19,20^.

Rainbow trout has a preference for temperatures between 10 and 15 °C, with an upper lethal limit of *ca.* 26 °C^21^. Here, fish were acclimated at 14 and 20 °C for 60 days before being exposed to a heat shock, with temperature increasing at a rate of 2 °C h^−1^ up to 30 °C. The supra-optimum acclimation temperature of 20 °C was used in this study hypothesising that this treatment could elicit natural protective mechanisms, helping the fish cope with the subsequent acute heat shock in comparison to fish acclimated at 14 °C. Moreover, our second hypothesis was that the antioxidant supplements would strengthen the capacity of fish to ameliorate the harmful effects of heat shocks. Accordingly, the present study evaluated the potential benefits of thermal acclimation and dietary provision of selenium yeast and chestnut polyphenol during heat stress by measuring survival rate, the expression of *HSP*s and *IL-1β* (interleukin-1β) genes, respiratory burst activity, lysozyme activity, and some biochemical and haematological factors including serum cortisol, total protein, albumin and red blood cells (RBC) in rainbow trout (*Oncorhynchus mykiss*) which is a widely cultured species in cold-freshwater systems.

## Materials and methods

### Experimental diets

Raw materials for the preparation of the experimental fish diets were purchased from 21 Beyza Feed Mill, Shiraz, Iran. Three iso-caloric, iso-lipid, and iso-nitrogenous diets were prepared using the same raw materials (Table 1) based on the NRC nutrient requirement for rainbow trout fingerlings, including a control diet which contained only the basal diet without any supplementation. The other two diets were supplemented with either selenium yeast or chestnut polyphenol as below. Selenium yeast (SelPlex®, Alltech, KY) at 5 mg/kg and chestnut polyphenol extract (Silvateam s.p.a., Italy) at 2 g/kg were each added to the other ingredients for their respective experimental diet and then mixed and prepared separately. Selenium is an essential microelement for fish but there is a narrow safety range between doses that lead to deficiency or toxicity. The selenium yeast concentration was chosen according to previous standard requirement recommendation which doses ≤ 15 mg/kg selenium per feed was considered as the safe and 3–5 mg/kg were the most recommended doses in salmonid diets^16,19,22^. The supplementary dose of chestnut polyphenol was also selected according to previous studies reporting the 2 g/kg as the most effective dose in fish^19,20^. For all three diets, pellets with a 2–2.5 mm in diameter and 3–5 mm in length (based on pelletizer features) were prepared, dried at 40 °C for 24 h and manually sieved to remove dust and stored at 4 °C until use. Fish were fed three times a day (i.e., 08:00, 13:30, and 16:00 h) at the rate of 3% of the total fish biomass in each tank.

**Table 1:** The ingredients of the experimental diets fed to rainbow trout during 60-day acclimation phase of the present study.

Feed Ingredient	Basal (control) diet*
Fish meal	485
Wheat meal	175
Wheat gluten	63.96
Fish oil	150
Soybean meal	100
Methionine	7.5
Lysine	5
Vitamin C	0.04
Organic zinc	1
Additives**	12.5
Antioxidant supplements	
Selenium diet (SelPlex®, Alltech, KY)	5
Polyphenol diet (Silvateam s.p.a., Italy)	2
Nutrient composition	
Crud protein	≥ 45%
Crud fat	≤ 14%
Fiber	≤ 2%
Moisture	≤ 10%
Phosphorous	≥ 0.8%
Calcium	1%
Magnesium	0.05%
Digestible energy	4300 kcal/Kg

*All the values are in g per kg of experimental diet except for SelPlex which is in mg/kg.

**Additives include: Vitamin A, Vitamin D, Vitamin E, Vitamin B (B1, B2, B5, B6, B12), Choline Chloride and minerals (Fe, Cu, Mn, and I) based on the NRC requirement for rainbow trout fingerlings.

### Fish rearing condition and sampling

A total of 756 rainbow trout fingerlings with a mean initial weight of 9.1 ± 0.4 g were provided by a local fish farm (Yasuj, Iran, natal temperature 13–16 °C) and transported to the aquaculture facility at Shiraz University. The fish handlings were conducted with minimal suffering of experimental animals based on the regulations of the Institutional Animal Care and Ethics Committee of Shiraz University in line with EU Directive 2010/63/EU for animal experiments.

Fish were randomly allocated into 18 indoor fiberglass tanks (cylinder-shaped 110 cm in diameter and 122 cm in height, ca. 900 l volume) supplied with thoroughly aerated well-water (14-16 ºC). Fish were acclimated for 20 days to laboratory conditions and during this period, they were fed with a basal diet (21 Beyza Feed Mill, Shiraz, Iran).

This study was conducted in two phases, including an acclimation phase and a heat shocks phase*:*

#### Acclimation phase

At the end of the initial 20 days of acclimation to laboratory conditions, the water supplied into nine tanks (as cold water category) was set to 14 °C, and for the rest nine tanks (as warm water category) was set to 20 °C (in 48 h, 2–3 °C increase per day based on previous research^7,23^ to minimize any stress).

The tanks were monitored every two hours for temperature (14 ± 0.5 °C and 20 ± 0.5 °C) as well as daily for pH and dissolved oxygen.

Treatments in the cold water category were as: (1) fish fed on a basal diet with no supplement (Cold-B), (2) fish fed on a diet supplemented with organic selenium (Cold-Se) and (3) fish fed on a diet supplemented with polyphenol (Cold-P). Similarly, treatments in the Warm water category were as: (4) fish fed on a basal diet with no supplement (Warm-B), (5) fish fed on a diet supplemented with organic selenium (Warm-Se), and (6) fish fed on a diet supplemented with polyphenol (Warm-P). Each treatment consisted of three replications, each of which involved 42 individuals.

#### Heat shocks phase

In this phase, after 60-day acclimation, fish in all treatments (in both cold and warm categories) were subjected to heat shocks up to 30 °C. To this end, an aquarium heater (RS 408-E-200 W, Risheng–China) was used to gradually increase the water temperature of all tanks up to 30 °C at a rate of 2 °C h^−1^.

The tanks were well aerated, and the behaviour of the fish was closely monitored during this period, and the timing of any mortalities was recorded. Fish were not fed during the heat shocks phase.

The survival rate (SUR) after heat shocks was calculated as below:

$${\text{Survival}}\;{\text{Rate}}\;\left( \% \right) = \left( {{\text{No}}.\;{\text{of}}\;{\text{fish}}\;{\text{after}}\;{\text{each}}\;{\text{heat}}\;{\text{shock}}/{\text{No}}.\;{\text{of}}\;{\text{fish}}\;{\text{at}}\;{\text{the}}\;{\text{end}}\;{\text{of}}\;{6}0\;{\text{days}}} \right) \times {1}00$$

At the end of the 60-day acclimation phase and during the heat shocks phase when the temperature of tanks reached 24, 28 and 30 °C, three fish per tank were sampled (nine fish at each sampling point from each treatment). At each sampling point, live fish were immediately anesthetized with clove powder (150 mg/l) and blood samples were collected with non-heparinized 2.5 ml plastic syringes (23-gauge needles) via the caudal vein. Serum was obtained by centrifuging blood samples at 6000 g for 15 min (K241R, Centurion, UK) and kept at − 80 °C for further analysis.

At the same time, whole liver tissue samples were taken and snap-frozen in liquid nitrogen. Samples were stored at − 80 °C until RNA extraction.

In addition, specific growth rate (SGR) and feed conversion ratio (FCR) were calculated at the end of the 60-day acclimation phase for the fish in each treatment as described in Islam et al.^3^ and Vazirzadeh et al^24^.

### Biochemical parameters

Serum total protein and albumin were measured by using commercial kits (Pars Azmun, Iran, Cod 96001-95003) according to manufacturers’ manuals. Total protein was measured by the Biuret method, with the Cu reagent at 546 nm. Albumin was measured through the selective interaction between Bromocresol Green (BCG) and albumin, resulting in the formation of a chromophore and colorimetric detection at 546 nm^24^.

### Serum cortisol

The serum cortisol level was measured using an ELISA commercial kit (DiaMetra, Italy, DKO 001) containing antigenic cortisol conjugated with horseradish peroxidase. During the assay, a blue colour complex was created that its intensity was proportional to the amount of cortisol in the sample. Optical absorption of the samples was read using a 490 and 630 nm wavelength in a plate reader (BioTek, UK).

### Immunological parameters

Respiratory burst activity was measured using nitro-blue tetrazolium (NBT)- a substrate internalized by macrophages from fresh blood. This assay was carried out as described in Vazirzadeh et al^24^. Briefly, 50 µl of blood was mixed with an equal volume of 0.2% NBT (Merck, Germany) solution and incubated for 30 min at 25 °C, then 50 µl of NBT and blood mixture was mixed with 1 ml dimethyl formamide and centrifuged (K241R, Centurion, UK) at 6000 g for 15 min. The absorbance of the supernatant was read at 540 nm using a spectrophotometer (PG Instruments Ltd, UK).

Lysozyme activity was also measured as described in Vazirzadeh et al^24^. Briefly, 10 µl of serum was added to 90 µl suspension of *Micrococcus luteus* cell wall (0.2 mg ml^−1^ in sodium phosphate buffer with pH 7.4), and the absorbance was measured at 450 nm after 0 and 10 min using a plate reader (BioTek, UK). The level of lysozyme activity (unit/min) was defined as the amount of enzyme that caused a decrease in absorbance of 0.001 per min.

### Haematological parameter

The RBC of fresh blood was assayed by Natt and Herrick's staining method, as detailed in Svobodová et al^25^. Briefly, 10 µl of fresh blood was mixed with 1990 µl of Natt and Herrick solution (Bioanalytic GmBH, Germany). Then 10 µl of this mixture was used for cell counts using a haemocytometer (Marien Feld, Germany) under a light microscope (Leica, Germany) with 40× magnification.

### RNA extraction and RT-qPCR

The total RNA was extracted from the liver using a whole RNA extraction column kit (DenaZist Asia, Iran, S-1020). RNA degradation and contamination with DNA were assayed on 1% agarose gel electrophoresis (Thermo Fisher Scientific, MA). The RNA concentration and purity were also checked using a NanoDrop Spectrophotometer (Thermo Fisher Scientific, MA). A total of 1 µg from each RNA sample was used to synthesize cDNA using a first strand cDNA synthesis kit (Sinnaclon, Iran, RT5201) according to the manufacturer's protocol and stored at − 80 °C until further use.

The gene expressions were quantified in StepOne™ Real-Time PCR (Thermo Fisher Scientific, MA) using 2 × SYBR Green (RealQ Plus Master Mix Green, Amplicon A/S, Denmark). The *β-actin* gene (181 bp) was used as the housekeeping gene to normalize the level of expression for *HSP70α*, *HSP70β*, *HSP90β*, and *IL-1β* in different samples amplified using the specific primers (Table 2). Reaction mixtures contained 5 µl of 2 × SYBR Green, 0.5 µM of each primer (forward and reverse), 1 µl of template cDNA, and 3 µl of distilled water in a final volume of 10 µl. For all PCR primer pairs, optimal annealing temperatures and reaction conditions were determined by examining the annealing temperature in gradient PCR using StepOne thermocycler (Thermo Fisher Scientific, MA). The cycling condition was 95 °C for 15 min, 95 °C for 15 s, 64 °C for 30 s, and 72 °C for 15 s. A melting curve analysis (95 °C for 5 s and 64 °C for 30 s) was performed after each qPCR run to verify the amplification specificity. The relative levels of *HSPs* and *IL-1β* mRNA were expressed using the 2^(−∆∆CT)^ method^26^.

**Table 2:** The Specifications and PCR product length for the primer pairs used in this study for genes expression analysis in rainbow trout.

Primer name	Primer sequence (5′–3′)	Ta (°C)	Amplification efficiency (%)	Product size (Bp)	AN
*β-actin*	F:CGAGACATCAAGGAGAAGC	64	95.38	181	XM_021595780
	R:CCATACCGAGGAAGGAGG				
*HSP70α*	F:GGCTCAGCAAAGAGGATATT	64	97.74	167	XM_036964716
	R:CTCCACGCTGCTCTTCATATT				
*HSP70β*	F:GGCTCAGCAAAGAGGATATT	64	96.86	167	NM_001124745
	R:CTCCACGCTGCTCTTCATATT				
*HSP90β*	F:TGCGCTACCACAGCTCTCAGT	64	96.56	183	NM_001124231
	R:GGTCCTTGCTCTCACCAGTGA				
*IL-1β*	F:TCTACCTGTCCTGCTCCAA	64	95.99	191	NM_001124347
	R:GTCCGTGCTGATGAACCA				

AN: NCBI GenBank accession number for the rainbow trout reference gene sequence used for the design of the primers.

### Statistical analyses

The normality and homogeneity of the data were tested using Shapiro–Wilk’s and Levene’s tests, respectively. Data were log (gene expression), or arcsine (survival rate) transformed to meet analyses of variance (ANOVA) assumptions of normality and homogeneity of variance if necessary. A two-way ANOVA was used to detect differences among treatments using the R version 3.6.3 software (R Core Team 2019). When ANOVA detected any differences among treatments, the means of treatments were compared using Tukey’s post hoc tests. The data are presented as least-squares means ± SEM (pooled standard error mean of the experiment). The significance level was considered at *P* ≤ 0.05. When the interaction of the Temperature × Supplement was significant, the main effects of treatments and supplements were not presented (Details of two-way ANOVA and corresponding P-values are presented in the Tables 3, 4).

**Table 3:** The results of two-way ANOVA comparing the effect of treatment and temperature and their interaction on growth performance, haemato-immunological and biochemical parameters of rainbow trout acclimated at 14 and 20 °C and fed on three dietary treatments (basal and supplemented with selenium yeast or polyphenol) for 60 days followed by thermal shocks up to 30 °C.

Parameter	Temperature	*F*-value			*P*-value		
		Temperature	Diet	Temperature × Diet	Temperature	Diet	Temperature × Diet
IBW^a^	After 60-day	0.39	1.18	0.23	0.55	0.36	0.79
FBW^b^	After 60-day	1.49	0.84	0.34	0.26	0.47	0.71
WG^c^	After 60-day	0.46	0.45	0.04	0.52	0.65	0.95
SGR^d^	After 60-day	0.50	0.44	0.05	0.50	0.66	0.94
FCR^e^	After 60-day	0.16	0.85	0.13	0.69	0.47	0.88
Survival rate (%)	After 60-day	571.6	1.7	1.4	0.28	0.28	0.28
	24 °C Shock	563.2	1.6	1.5	0.28	0.28	0.28
	28 °C Shock	583.2	1.4	1.4	< 0.00	0.28	0.28
	30 °C Shock	535.69	25.56	25.56	< 0.00	< 0.00	< 0.00
Total protein (g/dl)	After 60-day	9.43	6.31	3.39	0.00	0.50	0.08
	24 °C Shock	9.61	7.22	2.91	0.00	0.36	0.09
	28 °C Shock	9.84	0.67	1.46	0.00	0.53	0.04
	30 °C Shock	0.14	1.34	0.02	0.70	0.27	0.88
Albumin (g/dl)	After 60-day	18.42	4.47	3.38	0.00	0.08	0.04
	24 °C Shock	16.38	5.36	3.27	0.00	0.07	0.03
	28 °C Shock	0.36	0.003	0.13	0.55	0.99	0.87
	30 °C Shock	0.67	1.16	0.22	0.41	0.32	0.63
Respiratory burst activity (OD)	After 60-day	7.30	603.72	150.90	0.01	< 0.001	< 0.001
	24 °C Shock	0.04	539.45	4.09	0.83	< 0.001	0.02
	28 °C Shock	3.40	96.75	3.41	0.07	< 0.001	0.04
	30 °C Shock	1.64	1.61	3.55	0.21	0.22	0.07
Lysozyme (U/min)	After 60-day	1.74	1.93	0.49	0.19	0.16	0.61
	24 °C Shock	1.87	1.86	0.61	0.00	0.03	0.00
	28 °C Shock	1.81	1.78	0.47	0.01	0.02	0.00
	30 °C Shock	1.83	1.68	0.51	0.19	0.14	0.58
RBC (cell/mm^3^) (× 10^4^)^f^	After 60-day	12.38	13.18	23.36	0.00	0.00	0.00
	24 °C Shock	12.43	13.58	24.71	0.00	0.00	0.00
	28 °C Shock	13.01	12.87	23.21	0.00	0.00	0.00
	30 °C Shock	12.51	13.05	24.12	0.00	0.00	0.00
Cortisol (mcg/dL)	After 60-day	1.15	0.20	0.56	0.29	0.81	0.57
	24 °C Shock	2.11	1.31	0.87	0.03	0.04	0.00
	28 °C Shock	0.07	3.70	0.77	0.00	0.01	0.00
	30 °C Shock	0.95	0.84	0.61	0.00	0.00	0.00

^a^*IBW* Initial body weight (g).

^b^*FBW* Final body weight (g).

^c^*WG* Weight gain (%).

^d^*SGR* Specific growth rate (% day^−1^).

^e^*FCR* Feed conversion ratio.

^f^*RBC* Red blood cell.

**Table 4:** The results of two-way ANOVA comparing the effect of treatment and temperature and their interaction on *HSP70α*, *HSP70β*, *HSP90β* and IL-1β genes of rainbow trout acclimated at 14 and 20 °C and fed on three dietary treatments (basal and supplemented with selenium yeast or polyphenol) for 60 days followed by thermal shocks up to 30 °C.

Parameters	Temperature	*F*-value			*P*-value		
		Temperature	Diet	Temperature × Diet	Temperature	Diet	Temperature × Diet
*HSP70α*	After 60-day	18.89	21.12	10.19	0.004	0.001	0.01
	24 °C Shock	12.85	20.86	27.24	0.11	0.01	0.03
	28 °C Shock	138.64	44.51	40.84	< 0.001	< 0.001	< 0.001
	30 °C Shock	116.19	328.11	133.92	< 0.001	< 0.001	< 0.001
*HSP70β*	After 60-day	0.63	2.63	0.28	0.04	0.00	0.03
	24 °C Shock	2.97	1.67	0.50	0.01	0.00	0.00
	28 °C Shock	694.50	597.21	889.77	< 0.001	< 0.001	< 0.001
	30 °C Shock	33.01	1.93	26.66	0.002	0.03	0.003
*HSP90β*	After 60-day	77.23	2.42	2.71	< 0.12	0.16	0.14
	24 °C Shock	0.51	4.59	0.25	0.04	0.03	0.00
	28 °C Shock	2148.8	1291.5	562.3	< 0.001	< 0.001	< 0.001
	30 °C Shock	138.95	769.01	550.79	< 0.001	< 0.001	< 0.001
*IL-1β*	After 60-day	3.86	0.31	0.74	0.02	0.03	0.00
	24 °C Shock	2.36	50.46	88.34	0.03	< 0.001	< 0.001
	28 °C Shock	158.07	1.01	8.98	< 0.001	0.01	0.01
	30 °C Shock	6.92	62.76	68.24	0.04	< 0.001	< 0.001

### Ethics statement

The fish handlings were conducted with minimal suffering of experimental animals based on the regulations of the Institutional Animal Care and Ethics Committee of Shiraz University.

The experimental protocols were approved by the Animal Ethics Committee at Shiraz University. The experimental protocols (clinical examination, dissection, sampling, sample processing, microscopical examination, physiological and immunological analyses) were carried out in accordance with relevant guidelines and regulations supported by relevant references throughout the manuscript materials and methods section.

The current study was carried out in compliance with the ARRIVE guidelines when relevant methods were applied.

## Results

### Growth and survival

#### Acclimation phase

**Figure 1:**
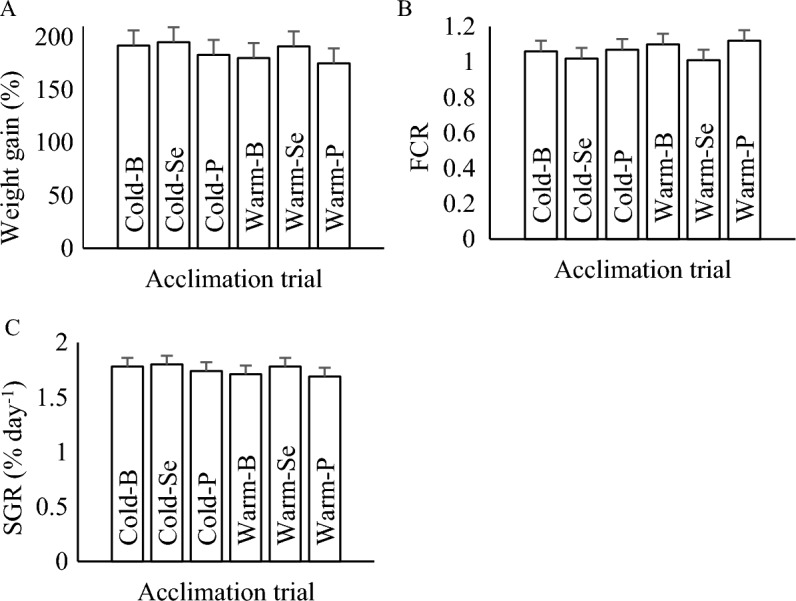
The effects of thermal acclimation and antioxidants supplemented diets on weight gain (**A**), specific growth rate (**B**) and feed conversion ratio (**C**) in rainbow trout fingerlings. No significant differences were observed among treatments.

At the end of the 60-day acclimation phase, although the fish in the cold category showed better growth performance, the acclimation temperature, supplemented diets, and their interaction did not significantly affect fish growth parameters, including weight gain (Fig. 1A), SGR (Fig. 1B), and FCR (Fig. 1C).

There were also no mortalities among the fingerlings during the acclimation period.

#### Heat shock phase

Subsequent to heat shocks, fish in the cold category (Cold-B, Cold-Se, and Cold-P treatments) began to show a sudden loss of balance upon reaching the temperature of 28 °C (Supplementary Fig. 1). Fish in all cold groups started to die after 28 °C heat shock. All fish in the Cold-Se treatment died upon reaching 29 °C and that’s why no sampling was possible for this treatment at 30 °C. In the other two Cold treatments (Cold- B and Cold-P) nearly 50% of the population died after 28 °C heat shock and the remaining died following 30 °C heat shock.

**Figure 2:**
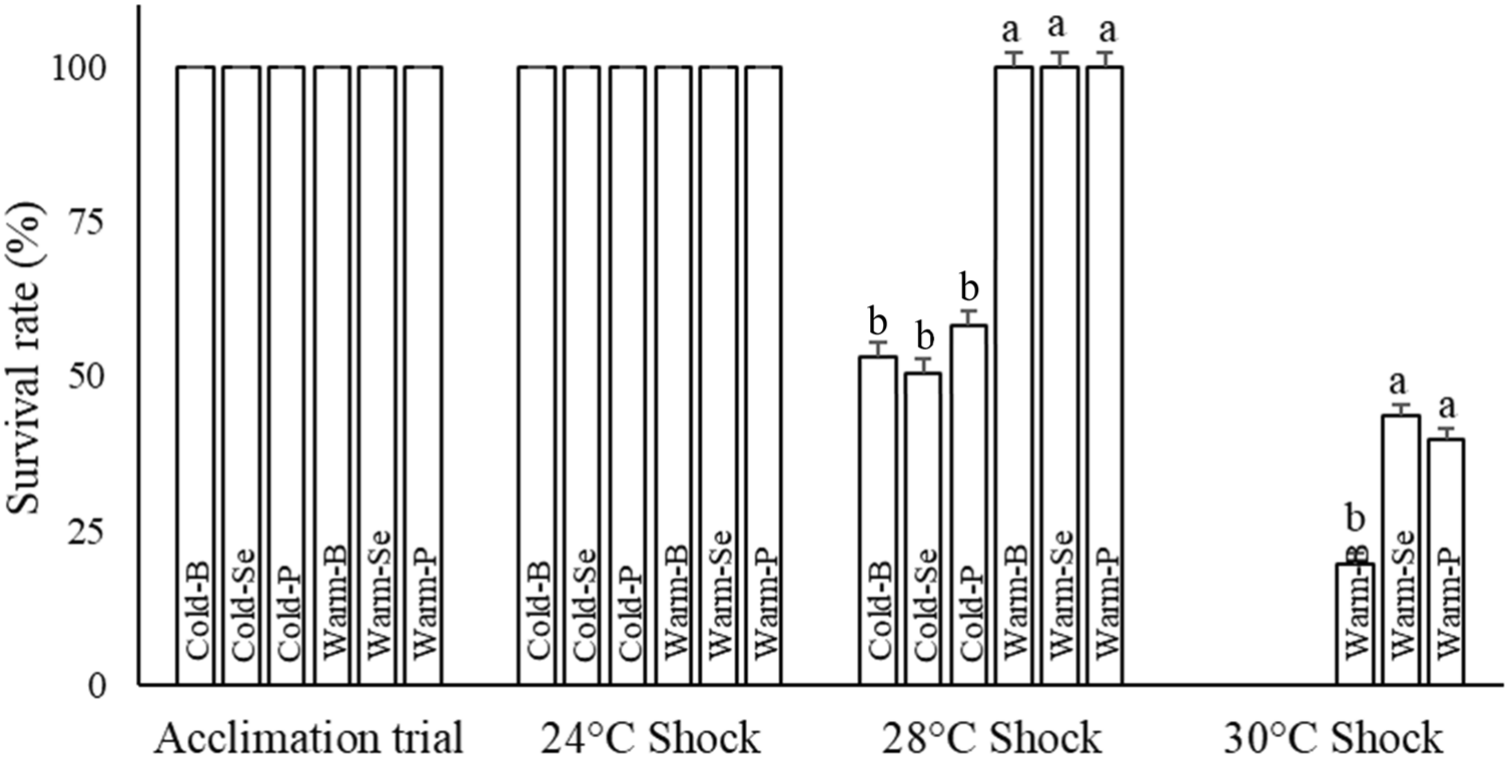
The cumulative survival rate of rainbow trout fingerlings during acclimation phase and during heat shocks. Means noted by a different letter indicate significant differences (*P* < 0.05) among treatments.

In contrast, no instance of mortality was observed in the warm category treatments until it reached 30 °C, and even after 30 °C heat shock, a subset of the population was alive. At the end of the heat shocks trial, the survival rate of fish in Warm-Se (43.5%) and Warm- P (39.8%) treatments were significantly higher than those fish in Warm- B (20%) (Fig. 2).

### Blood biochemistry and haematology

#### Total protein

**Figure 3:**
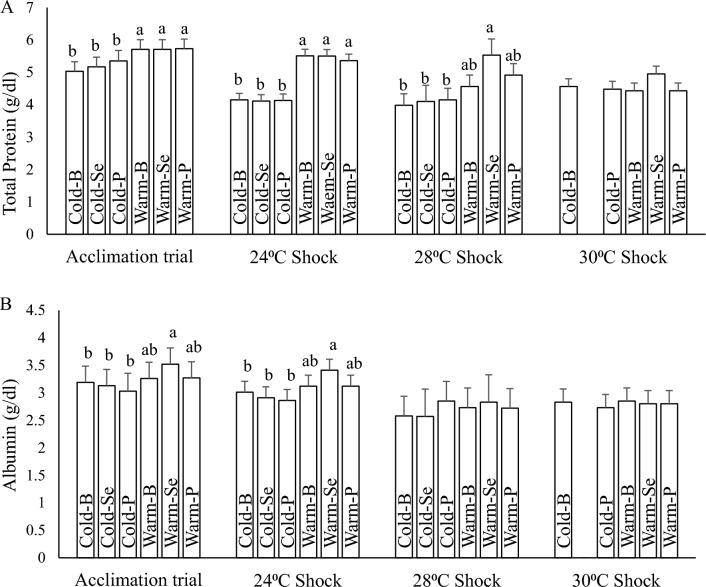
Serum total protein (**A**) and albumin (**B**) of rainbow trout fingerlings after the 60 days acclimation period and during heat shocks. Means noted by a different letter indicate significant differences (*P* < 0.05) among treatments.

After the 60-day acclimation period, the serum total protein level was higher in the warm water category (treatments acclimated to warm temperature) in comparison to the cold water category (cold water acclimated fishes) (Fig. 3A). Following the 24 °C heat shock, again the warm water acclimated fish showed a higher level of total serum protein. Interactive analysis of temperature and diet effects after 28 °C heat shock showed that fish in Warm-Se had significantly higher serum protein level than other treatments (Fig. 3A). Following the 30 °C heat shock, all fish showed similar total protein levels (Fig. 3A).

#### Albumin

The results of the serum albumin essay showed that fish in Warm-Se had higher serum albumin levels at the end of the 60-day acclimation period as well as following the 24 °C heat shock trial (Fig. 3B). There were no significant differences in albumin levels in fish exposed to heat shocks at 28 and 30 °C, regardless of their acclimation and feeding history (Fig. 3B).

#### Respiratory burst activity

**Figure 4:**
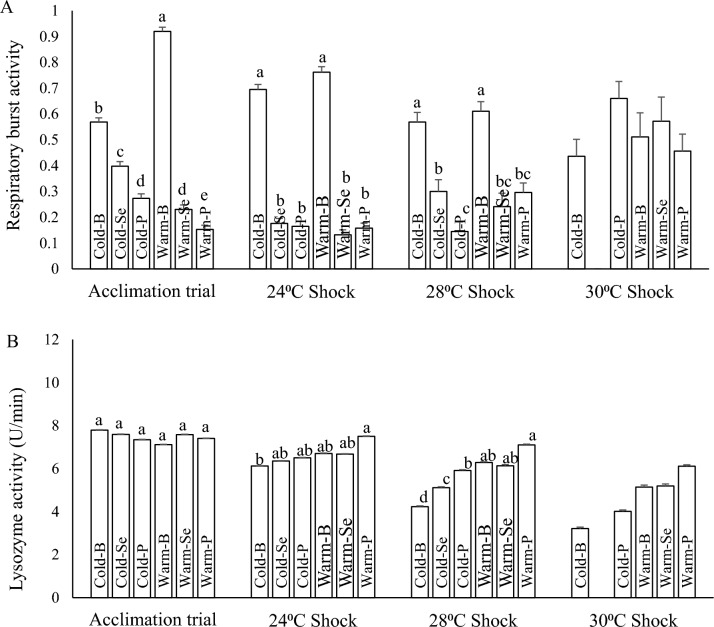
The respiratory burst (**A**) and lysozyme (**B**) activities of rainbow trout fingerlings after 60 days acclimation period and during thermal shocks. Means noted by a different letter indicate significant differences (*P* < 0.05) among treatments.

At the end of the 60-day acclimation period, diet significantly affected respiratory burst activity, and the fish fed on selenium and polyphenol supplemented diets in both the cold and warm categories showed lower respiratory burst activity in comparison to fish fed on the basal diet (Fig. 4A). Moreover, selenium and polyphenol supplementations significantly improved (decreased) respiratory burst activity compared to fish that received the basal diet during 24 and 28 °C heat shocks in both warm and cold groups (Fig. 4A).

#### Lysozyme activity

At the end of the 60-day acclimation trial, no significant differences were found in the lysozyme activity of fish in different treatments. However, following 24 °C heat shock, the lysozyme activity of fish in Warm-P group was significantly higher than those in the cold water category. The difference between the cold and warm acclimated fish was increased following the 28 °C heat shock, while all fish in the warm category except Cold-P showed significantly higher lysozyme levels than those fish in the cold category. A similar figure was observed in fish exposed to 30 °C heat shock. It’s important to note that lysozyme activity was gradually decreased in all treatments by increasing temperature (Fig. 4B).

#### Red blood cell count

**Figure 5:**
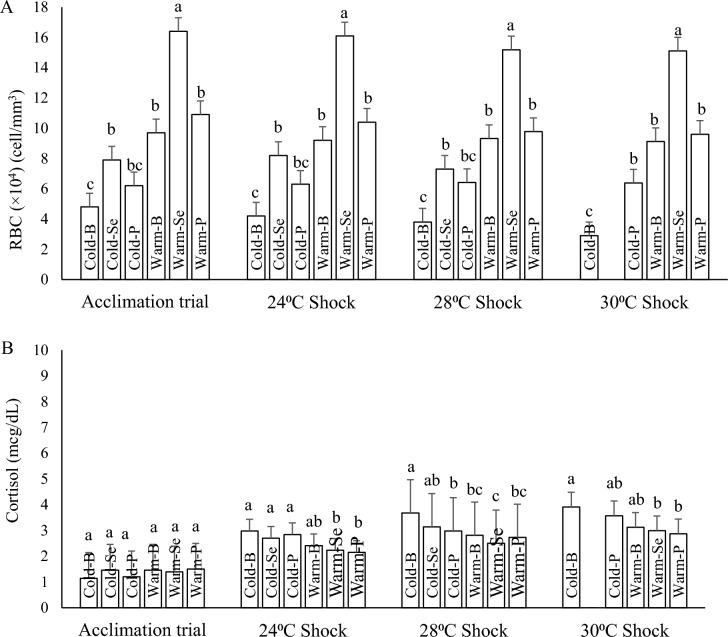
The effects of thermal acclimation and antioxidants supplemented diets on red blood cell count (**A**) and serum cortisol level (**B**) of rainbow trout fingerlings after 60 days acclimation period and during heat shocks. Means noted by a different letter indicate significant differences (*P* < 0.05) among treatments.

Fish in Warm-Se treatment showed the highest RBC (16.4 × 10^4^) at the end of the 60-day acclimation trial and during different heat shock temperatures. Moreover, fish in all treatments acclimated to warm water or received supplemented diets showed higher and significant RBC than those in Cold-B treatment (RBC = 4.8 × 10^4^) (Fig. 5A).

#### Cortisol

No significant differences were found in the basal cortisol concentrations measured at the end of the 60-day acclimation phase among treatments. However, an increase in cortisol levels was observed following the exposure of fish to 24 °C: The highest values were found in Cold-B fish, followed by fish in Cold-P, and Cold-Se treatments, whereas the lowest values were found in fish in Warm-Se and Warm-P treatments. Following 28 °C heat shock, a remarkable difference was observed in cortisol levels of treatments, and the highest significant cortisol level was recorded in fish acclimated at cold water without receiving any supplements (Cold-B). Although the fish in cold category still showed higher cortisol level after the 30 °C heat shock, the differences between cold and warm categories were lower than 28 °C, due to significant rise in the cortisol level of warm water acclimated fish (Fig. 5B).

### Gene expression

#### HSP genes expression

**Figure 6:**
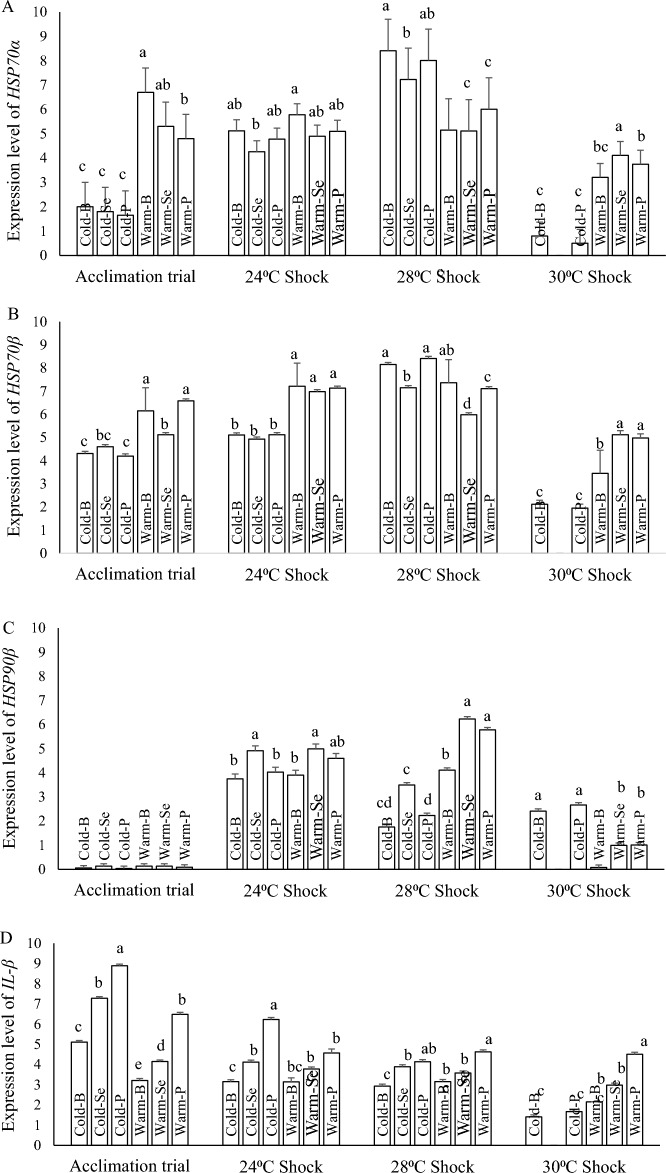
The expression of *HSP70α* (**A**), *HSP70β* (**B**), *HSP90β* (**C**) and *IL-1β* (**D**) of rainbow trout fingerlings after 60 days acclimation period and during heat shocks. Means noted by a different letter indicate significant differences (*P* < 0.05) among treatments.

The results showed that at the end of the 60-day acclimation phase, fish in all cold water categories (Cold-B, Cold-Se and Cold-P) had a significantly lower expression level of *HSP70α* in comparison to the warm water category (Warm-B, Warm-Se and Warm-P), but during heat shock phase, the expression level of *HSP70α* was gradually increased in cold water category, so that following 28 °C heat shocks, the *HSP70α* expression level was significantly higher in all three cold water adapted treatments in comparison to warm water reared fish. Surprisingly, after 30 °C heat shock, the expression level of both groups was suddenly dropped down. In this temperature, the warm water acclimated fish showed a higher expression level of *HSP70α* (Fig. 6A).

*HSP70β* showed the same expression trend as to *HSP70α* but in a lower increase rate. At the end of the acclimation trial and following the 24 °C heat shock, fish in the warm water category showed higher expression levels of *HSP70β,* but following the 28 °C heat shock, the expression level of *HSP70β* suddenly surged in cold water adapted fish that were significantly higher than those in warm water acclimated fish. Same as to *HSP70α*, again, fish in the warm water group showed higher expression level of *HSP70β* after 30 °C heat shock (Fig. 6B).

At the end of the 60-day acclimation trial, all groups showed low expression level of *HSP90β* and there were no significant differences among treatments (Fig. 6C). Exposure to heat shocks rapidly increased the expression level of *HSP90β* but again there were no significant differences between cold and warm categories after 24 °C heat shock, although the fish fed on selenium supplemented diets showed highest expression level of the gene in both cold and warm categories. Exposure to 28 °C heat shock resulted in significantly higher expression level of *HSP90β* in warm water acclimated treatments, and again fish received supplemented diets had higher expression levels (Warm-Se and Warm-P). In contrast to *HSP70α* and *HSP70β*, after the 30 °C heat shock, the warm water adapted fish showed lower expression level of *HSP90β* in comparison with fish acclimated at cold condition (*P* < 0.05) (Fig. 6C).

#### IL-1β gene expression

The results of the 60-day acclimation trial showed that acclimation of fish to warm water resulted in a lower expression level of *IL-1β,* although antioxidant feeding improved the immune status of fish but not at such a level of fish adapted to cold water*.* After 24 °C heat shock a significant decrease in *IL-1β* expression level was observed in cold water acclimated fish. Continued heat shock up to 28 °C heat shock, led to more decrease in the expression level of *IL-1β* in all cold water adapted groups, whereas there were no significant differences among cold and warm categories after 28 °C heat shock. Exposure to 30 °C heat shock led to a further decrease of *IL-1β* expression level in cold water acclimated groups, even though the expression level was lower than those in warm water acclimated groups (*P* < 0.05) (Fig. 6D).

## Discussion

Water temperature plays a significant role in the growth and metabolism of fish subjected to environmental temperature fluctuations. Sudden changes in supra-optimal temperature or heat shock affect key physiological functions, and understanding its effect on fish is of great importance for preparing to cope with climatic changes in the aquaculture industry^3,5,6,27–31^. The present study investigated the thermal acclimation and the provision of two dietary antioxidants on the tolerance of rainbow trout fingerlings to heat shocks.

Although fish kept at the higher temperature showed lower SGR and higher FCR rates, the overall growth rates were not significantly affected by Cold or Warm treatments, indicating that the upper threshold for growth of rainbow trout is around 20 °C. Within an individual lifetime, its performance such as growth rate, can be altered through acclimation to environmental temperature, exhibiting a phenotypic plasticity pattern. These results align with a study on chinook salmon (*Oncorhynchus tshawytscha*) growth performance metrics during a three-month thermal challenge at 19–20 °C^32^. Schram et al^33^. reported that the temperature preferred by Dover Sole (*Solea solea*) increases with increasing acclimation temperature and exceeds the optimal growth temperature. Similarly, the plasticity effect of temperature on growth rate and heat resistance was reported in *Schizothorax kozlovi*^34^.

Acclimation to a higher temperature (20 °C) for 60 days combined with dietary antioxidants found to enhance the survival rate of rainbow trout when exposed to heat shocks. The supra-optimal temperature acclimation was more effective in survival than supplements. This finding aligns with a range of studies in other species including rainbow trout^7,35^, Atlantic salmon^9^, stripped bass^36^, European seabass^3^, and aquatic invertebrates^30^. For instance, higher rates of survival were observed in European seabass reared at 16 °C and 24 °C compared to 8 °C and 32 °C and temperature ranges between 16 and 24 °C provided optimum conditions for high survival and good growth performance^3^. Furthermore, Jiang et al.^7^ found that the temperature preferred by rainbow trout increases with acclimation temperature and exceeds the optimal growth temperature. Their results showed that the heat tolerance of the rainbow trout after acclimation to a high temperature (22 °C) was significantly improved when the fish were challenged with a high temperature (20 °C) again. The juvenile rainbow trout acclimated for 9 days had the highest survival rate in comparison with the fish that experienced 0 d, 3d, and 6d acclimation.

The physiological and behavioral response of fish to thermal stress is related to underlying biochemical and molecular responses or changes in gene expression levels by the antioxidant defence system^3,27–29,31^ discussed hereafter.

Blood biochemical factors can be used as feasible physiological indices to recognize possible alterations in the organism exposed to stressful conditions^37^. Increase in total protein and albumin is related to a faster metabolism and stronger immune response in rainbow trout^24^. The higher level of serum protein in warm acclimated fish in this study was in agreement with a previous thermal acclimation study on *Abudefduf saxatilis* and *Scartella cristata* which showed a considerable increase in the total protein level at elevated temperatures^29^. Upregulated transcription of genes involved in protein synthesis or metabolism and acute-phase protein levels have previously been established as a key thermal stress response in Chinook salmon^32,38^; our results complement these findings at the functional total protein level. The higher levels of total protein observed in treatments receiving organic selenium supplement in 28 °C shock suggests fish’s ability to maintain the homeostasis of total protein content against environmental stress^22^. The organic selenium in the fish diet also increased albumin production in the liver at 20 °C, which aligns with the results of other studies^39–41^. A higher level of selenium micronutrient also plays a vital role in the stability of proteins^12,17^. The reduction in total protein levels at the end of thermal shock may also indicate an increase in anaerobic metabolism and protein catabolism, which may predispose fish to death^31,32^.

Heat shock is among the main stressors affecting the antioxidant defense system by production of reactive oxygen species (ROS) in key tissues of vertebrates^3,10,29,42^. The lower respiratory burst activity in treatments received dietary selenium yeast and polyphenol during the heat shocks phase in both acclimation temperatures indicates the positive effect of selenium yeast and polyphenol treatments on improving fish antioxidant defense and immune systems. The polyphenol effect on decreasing ROS is due to its phenolics and flavonoid content^19^. Further, the same level of respiratory burst activity in all treatments at 30 °C -which is the lethal temperature for rainbow trout-, suggests that immune and oxidative stress responses were collapsed at this temperature.

Lysozyme activity analyses showed that both thermal acclimation and antioxidants improved fish serum lysozyme activity following the exposure to heat shocks, while at the end of the 60-day acclimation trial, all treatments had the same lysozyme levels. The fish in Cold-B group had lower lysozyme activity during the heat shocks phase. Taking all together, it can be postulated that the culture history temperature as well as the dietary ingredients have crucial effects on the resilience of fish in stressful condition, what has already been reported in other species^23^.

With the acclimation of fish to a higher temperature, the number of red blood cells increased so that fish acclimated at warm water and fed on the selenium-supplemented diet (Warm-Se) had the highest RBC. In agreement with other studies, dissolved oxygen holding capacity decrease in elevated water temperatures leads to increase in red blood cells to compensate the oxygen-carrying capacity of blood in rainbow trout^43,44^, European seabass^3^,carp^20^, meagre^41^ and channel catfish^39^.

The survival rate of fish acclimated to warm water during the heat shock phase can potentially be explained by this change in red blood cells. Although the oxygen level for fish was kept at saturation level throughout the study, the combination of elevated water temperatures and decreasing the oxygen carrying capacity at higher temperature poses physiological challenges for rainbow trout survival. With temperature increase, the demand from fish for oxygen rises whereas the available oxygen in water has already decreased. Here, rainbow trout might compensate for this effect by increasing their red blood cell production in order to boost oxygen delivery to tissues. However, this strategy will not work beyond a critical temperature, and therefore, mortality in rainbow trout will occur by combination of hypoxia and heat stress compromising biological integrity.

Blood cortisol level serve as a proper indicator to measure the severity of environmental stress. The result of this current study showed that the cortisol level in rainbow trout after the 60-day acclimation trial was low regardless of temperature and diet. The lack of any significant difference in cortisol level after acclimation to different temperatures suggests that fish in this study could well tolerate and adapt to high temperature (20 °C). This means the upper thermal limit of rainbow trout can be extended with acclimation to a higher temperature or dietary conditions. In chinook salmon (*O. tshawytscha*) thermal shock (21.6 °C) increased cortisol levels slightly^45^. During heat shocks phase, the cortisol levels of all treatments increased up to 2–4 folds, but warm water acclimated fish and those received dietary antioxidants showed lower cortisol levels. This is another evidence supporting the positive effects of acclimation to higher temperature and dietary antioxidant provision in the enhancement of fish capacity to cope with heat shocks.

Acclimation to warm temperature has increased the *HSPs* expression threshold and subsequently caused higher thermal tolerance in several fish species^3,4,10,11,35,36^. The molecular investigation of thermal acclimation in this present study showed that acclimation to a higher temperature in combination with feeding on selenium and polyphenol led to significantly higher expression of *HSP70α*, *HSP70β* and *HSP90β* mRNAs of rainbow trout fingerlings at the end of 60-day acclimation period but resulted to lower expression of above-mentioned genes following the heat shocks. Taking all these results together may indicate that increased levels of basal *HSPs* increase whole-body thermal responses such as CT*max* and protect against the harmful effects of elevated temperature stress by restoring the thermally damaged proteins and preventing cytotoxicity^4,43^.

At the 30 °C shock, fish were likely in such a poor condition that the *HSP* expression was down-regulated again or suppressed in favor to conserve energy for basic physiological functions and ensure survival^38^. Our results were consistent with previous studies on rainbow trout^21^, lake trout (*Salvelinus namaycush*)^2^ and sea urchin (*Evechinus chloroticus*)^30^. Jiang et al.^7^ also reported that acclimation of rainbow trout juveniles to a high temperature (22 °C) over a longer period (9 d) lowered the expression of *HSP* genes compared to fish experienced shorter acclimation time (0–6 days). They reported that the expression level of *HSP* genes in fish acclimated to high temperature for a 9-days period was the same as to fish kept in optimum water temperature (16 °C). These results demonstrate the capacity of rainbow trout to tolerate higher ambient temperature when acclimated to high temperature for a sufficient period of time. This also reflected the genetic level that heat acclimation can improve the tolerance of rainbow trout to high temperature.

The extent of change in biochemical parameters and gene expression levels by heat shock in this study depended on acclimation temperature and dietary antioxidants. It is postulated that ROS production increases under heat stress^3,10,29^ and therefore, antioxidants and *HSP70* activity are required for protection against adverse effects of ROS. The increase in temperature and subsequent denaturation of proteins results in the generation of ROS^42^, which in turn leads to the overexpression of heat shock proteins (HSPs) to facilitate protein restoration and renaturation.

The results of this study clearly showed that acclimation of rainbow trout at high temperature can increase the capacity of fish to cope with higher heat shocks during sudden increase in environmental temperature. Antioxidant supplementation would also strengthen this capacity. A recent study on European seabass has also shown the ameliorative potential of dietary antioxidants (including vitamin C, E, β-glucan, and phycocyanin) against prolonged heat shocks^23^.

Fish acclimated to cold water showed higher *IL-1β* expression during acclimation phase, but warm water acclimated fish showed higher *IL-1β* expression during the heat shock phase. These results indicate that fish adapted to cold water have higher immune levels in normal conditions, but warm water acclimated fish have higher immune status during environmental shocks, a phenomenon has already been reported in different species^7^. The gradual decrease of *IL-1β* expression during heat shock phase may indicate that the capacity of fish to maintain normal immune status decrease under thermal shock above the fish normal tolerance range.

## Conclusion

Acclimation to a higher temperature combined with dietary selenium and polyphenol was effective on the rainbow trout's heat tolerance. Therefore, thermal acclimation and an increase in dietary antioxidant levels can be considered as a management strategy for climate change-caused thermal shock in trout aquaculture. The higher survival rate in high temperature acclimated and antioxidant received groups and its correlation to HSP genes were notable.

### Supplementary Information

Supplementary Information.

## Data Availability

All data generated or analyzed during this study are included in this article.
