# Supplementary material for: Acclimation to higher temperature and antioxidant supplemented diets improved rainbow trout (Oncorhynchus mykiss) resilience to heatwaves
Source: Sci Rep. 2024 May 18;14:11375. doi: 10.1038/s41598-024-62130-y (PMC11102425; doi:10.1038/s41598-024-62130-y)

Supplementary Figure 1: A photograph of rainbow trout observations at the beginning and during the heat shock trial.

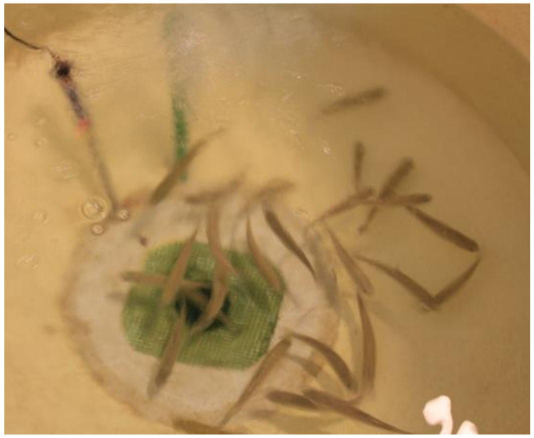

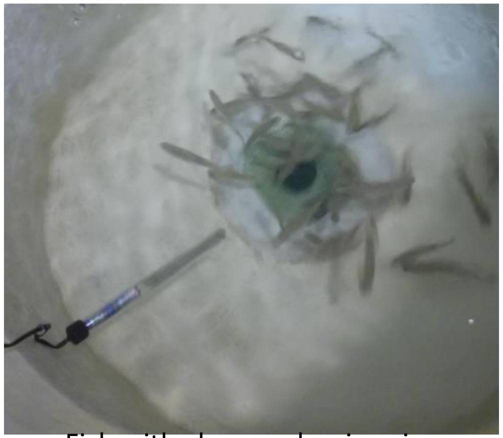

fish with normal swimming
at the beginning of heat shock

Fish with abnormal swimming
and loss of equilibrium at the
beginning of heat shock

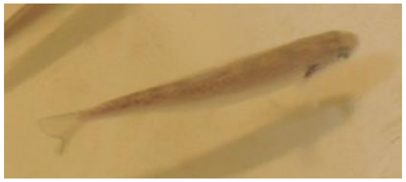

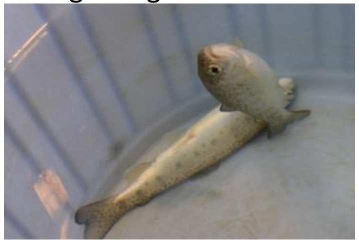

Supplement: Supplementary file 1 — Supplementary Information. [file 41598_2024_62130_MOESM1_ESM.pdf]
